# Epidermal Growth Factor Receptor as Target for Perioperative Elimination of Circulating Colorectal Cancer Cells

**DOI:** 10.1155/2022/3577928

**Published:** 2022-01-07

**Authors:** Mandy Gruijs, Rens Braster, Marije B. Overdijk, Tessa Hellingman, Sandra Verploegen, Rianne Korthouwer, Berend J. van der Wilk, Paul W. H. I. Parren, Hans J. van der Vliet, Marijn Bögels, Marjolein van Egmond

**Affiliations:** ^1^Amsterdam UMC, Vrije Universiteit Amsterdam, Department of Molecular Cell Biology and Immunology, Cancer Center Amsterdam–Amsterdam Infection and Immunity Institute, De Boelelaan 1117, Amsterdam, Netherlands; ^2^Genmab, Uppsalalaan 15, Utrecht, Netherlands; ^3^Amsterdam UMC, Vrije Universiteit Amsterdam, Department of Surgery, Cancer Center Amsterdam, De Boelelaan 1117, Amsterdam, Netherlands; ^4^Leiden University Medical Center, Department of Immunohematology and Blood Transfusion, Albinusdreef 2, Leiden, Netherlands; ^5^Lava Therapeutics, Yalelaan 60, Utrecht, Netherlands; ^6^Amsterdam UMC, Vrije Universiteit Amsterdam, Department of Medical Oncology, Cancer Center Amsterdam, De Boelelaan 1117, Amsterdam, Netherlands

## Abstract

Surgical resection of the tumor is the primary treatment of colorectal cancer patients. However, we previously demonstrated that abdominal surgery promotes the adherence of circulating tumor cells (CTC) in the liver and subsequent liver metastasis development. Importantly, preoperative treatment with specific tumor-targeting monoclonal antibodies (mAb) prevented surgery-induced liver metastasis development in rats. This study investigated whether the epidermal growth factor receptor (EGFR) represents a suitable target for preoperative antibody treatment of colorectal cancer patients undergoing surgery. The majority of patients with resectable colorectal liver metastases were shown to have EGFR + CTCs. Three different anti-EGFR mAbs (cetuximab, zalutumumab, and panitumumab) were equally efficient in the opsonization of tumor cell lines. Additionally, all three mAbs induced antibody-dependent cellular phagocytosis (ADCP) of tumor cells by macrophages at low antibody concentrations *in vitro*, independent of mutations in EGFR signaling pathways. The plasma of cetuximab-treated patients efficiently opsonized tumor cells *ex vivo* and induced phagocytosis. Furthermore, neither proliferation nor migration of epithelial cells was affected *in vitro*, supporting that wound healing will not be hampered by treatment with low anti-EGFR mAb concentrations. These data support the use of a low dose of anti-EGFR mAbs prior to resection of the tumor to eliminate CTCs without interfering with the healing of the anastomosis. Ultimately, this may reduce the risk of metastasis development, consequently improving long-term patient outcome significantly.

## 1. Introduction

Colorectal cancer is one of the most frequent types of cancer worldwide. Annually, approximately 1.9 million patients are diagnosed with colorectal cancer globally, and more than 935,000 patients die of this disease [1]. Most patients require surgery, as resection of the primary tumor is the cornerstone of treatment, which still provides the best curative option [2–4]. Nonetheless, the development of liver metastases is frequently observed and associated with high morbidity and mortality [5]. The prognosis of patients with colorectal liver metastases is poor, with a median survival time of eight months without treatment, and 5-year survival rates of 15–50%, mainly depending on the presence of extrahepatic disease [6]. Patients with metastatic colorectal cancer are often eligible for palliative chemotherapy, consisting of a combination of 5-fluorouracil, oxaliplatin, irinotecan, antibodies against epidermal growth factor receptor (EGFR) and/or vascular endothelial growth factor (VEGF), and regorafenib [7].

Up to 75% of colorectal cancer patients have disseminated circulating tumor cells (CTC) in their blood [8, 9]. Moreover, an increase in CTC numbers can be observed shortly after surgery, suggesting shedding of tumor cells due to manipulation of the tumor during surgery [10]. The presence of CTCs is correlated with poor survival of both patients with primary colorectal cancer and patients with resectable liver metastases [8, 11–13]. Additionally, high postoperative levels of CTCs predicted tumor recurrence [11, 12].

We and others previously demonstrated that surgery enhances the risk of liver metastasis development in animal models [14–16]. Inflammatory responses due to abdominal surgery-induced changes in the liver vasculature, which enhanced adhesion of CTCs and subsequent outgrowth of liver metastases [15, 17, 18]. Thus, even though colorectal surgery is the mandatory first line of treatment to remove the bulk of the tumor load, this may paradoxically create a niche that allows adhesion of CTCs and subsequent liver metastasis development in patients. Taken together, the perioperative period can have a substantial impact on clinical outcomes. Several strategies for perioperative treatment have been proposed to minimize surgery-induced cancer progression [19–22]. However, it has been reported that perioperative chemotherapy has no significant effect on overall survival [23]. Therefore, other types of therapy should be exploited for perioperative treatment.

Currently, research focuses on the use of targeted treatment in colorectal cancer. For instance, patients with metastatic colorectal cancer expressing EGFR can be treated with anti-EGFR monoclonal antibodies (mAb). These mAbs block the ligand binding site of this receptor, preventing the binding of epidermal growth factor (EGF) and subsequent signaling [24, 25]. Approximately 80% of colorectal carcinomas show overexpression of EGFR [26–28]. However, mutations in EGFR signaling pathways, which result in continuous signaling—irrespective of ligand binding—are frequently observed and render antibody treatment less effective. Nevertheless, in addition to the direct effect on signaling, mAbs can induce Fc-mediated effector functions [29]. After binding of an antibody to both its antigen expressed on the surface of a target cell via the Fab domain and an effector cell by the association of the Fc domain to an Fc-receptor, antibody-dependent cellular cytotoxicity (ADCC) or antibody-dependent cellular phagocytosis (ADCP) may be induced to eliminate the target cell. These effector functions are independent of mutations in the signaling pathways.

Our hypothesis is that elimination of CTCs by means of antibody treatment may reduce liver metastasis development and improve overall patient survival. We previously showed that preoperative treatment with antibodies directed against tumor cells prevented surgery-induced liver metastasis development in rats [30]. The therapeutic effect was mainly mediated by potent ADCP induced by Kupffer cells (macrophages residing in the liver) and monocytes [30–32]. Although the potential of perioperative antibody treatment to prevent metastasis development has not been investigated extensively in the clinic [33], one study showed increased 7-year survival and reduced overall mortality in patients with primary colorectal cancer, who were treated postoperatively with anti-EpCAM antibodies [34]. Treatment had no effect on the local recurrence of the primary tumor but reduced the occurrence of distant metastases in approximately 30% of the patients. However, we anticipate that preoperative treatment could be more suitable to eliminate tumor cells that have disseminated during surgery. Recently, a study has started to determine the safety and efficacy of preoperative treatment with the anti-CA 19–9 mAb MVT-5873 in patients undergoing resections for pancreatic cancer, cholangiocarcinoma, or metastatic colorectal liver metastases. However, results have not been reported yet [35]. We previously demonstrated that anti-EGFR mAb treatment results in efficient elimination of CTCs in animal models [31, 32]. Moreover, EGFR is often highly overexpressed on human tumor cells. Combined with the fact that anti-EGFR mAbs are already clinically used to treat metastasized colorectal cancer, we hypothesize that EGFR also represents an appropriate target for preoperative antibody treatment.

This study aimed to investigate the suitability of the anti-EGFR mAbs cetuximab (Erbitux; mouse/human chimeric IgG1 antibody), zalutumumab (human IgG1 antibody), and panitumumab (Vectibix; human IgG2 antibody) for preoperative treatment to eliminate CTCs in patients, which may prevent surgery-induced metastasis development.

## 2. Methods

### 2.1. Antibodies and Plasma

#### 2.1.1. Antibodies

The therapeutic anti-EGFR mAbs cetuximab and panitumumab were purchased from Merck (Schiphol-Rijk, the Netherlands) and Amgen (Breda, the Netherlands), respectively. Zalutumumab (HuMax-EGFR, clone 2F8) was generated by Genmab (Utrecht, the Netherlands) as described previously [36].

#### 2.1.2. Plasma

Plasma from 10 metastatic KRAS wild-type colorectal cancer patients (mean age: 60.6 years (50–73 years), 6 males) previously treated with and progressive after systemic palliative chemotherapy was obtained before and four hours after an initial dose of cetuximab (500 mg/m^2^ i.v.), administered as part of the COLOCETUX trial (NCT01691391) [37]. The trial was approved by the medical ethical committee of the Amsterdam UMC, location VUmc. All patients signed informed consent according to Dutch and international law.

### 2.2. Cell Culture

#### 2.2.1. Tumor Cells

The human epidermoid carcinoma cell line A431 and the human colorectal carcinoma cell lines Caco2, HCT116, HT29, RKO, SW620 and SW948 (ATCC, Manassas, VA) were cultured in DMEM (Invitrogen, Paisley, UK) supplemented with 10% heat-inactivated fetal calf serum (FCS), 100 U/ml penicillin, 100 *μ*g/ml streptomycin, and 200 *μ*ML-glutamine (hereafter referred to as complete DMEM) under standard incubator conditions (37°C, 5% CO_2_). Cell suspensions were prepared by enzymatic digestion using trypsin-EDTA solution (Invitrogen). Viability was assessed by trypan blue exclusion and always exceeded 95%.

#### 2.2.2. Human Macrophages and Natural Killer Cells

Human monocytes or natural killer (NK) cells were isolated from buffy coats (Sanquin, Amsterdam, the Netherlands) from healthy blood donors <24 h after blood collection. All donors gave informed consent. Whole blood was diluted 1 : 1 in PBS and loaded on Lymphoprep (Nyegaard, Oslo, Norway), whereafter cells were separated by density centrifugation. Peripheral blood mononuclear cells (PBMC) were extracted from the interphase of the Lymphoprep gradient and washed three times with PBS supplemented with autologous serum. Either CD14+ monocytes or NK cells were isolated from the PBMC fraction with cell separation beads (positive selection for CD14+ monocytes, negative selection for NK cells) (Miltenyi Biotech, Leiden, the Netherlands), according to the manufacturer's protocol. Isolated cells were washed and resuspended in RPMI 1640 (Invitrogen) supplemented with 10% heat-inactivated FCS, 100 U/ml penicillin, 100 *μ*g/ml streptomycin, and 200 *μ*ML glutamine (hereafter referred to as complete RPMI). CD14+ monocytes were cultured in complete RPMI with 50 ng/ml macrophage colony-stimulating factor (M-CSF) (eBioscience, San Diego, CA) for eight days.

### 2.3. Detection of CTCs

Whole blood (7.5 ml per sample), either from healthy donors spiked with defined amounts of human carcinoma cell lines or from patients with metastatic colorectal cancer, was diluted 1 : 1 in PBS and loaded on Lymphoprep, thereafter cells were separated by density centrifugation. PBMCs were extracted from the interphase of the Lymphoprep gradient and washed three times with PBS supplemented with autologous serum. EpCAM + tumor cells were isolated from the PBMC fraction with cell separation beads (positive selection) (Miltenyi Biotech), according to the manufacturer's protocol. Isolated cells were washed and stained with PerCP-Cy5.5-labelled anti-human EpCAM antibody (clone EBA-1, BD Biosciences, San Jose, CA) and PE-labelled anti-human EGFR antibody (clone AY13, BioLegend, San Diego, CA) and analyzed by flow cytometry (LSR Fortessa X20, BD Biosciences). CTCs were defined as EpCAM + EGFR + cells within the live cell population based on FSC-SSC gating.

### 2.4. Flow Cytometry

#### 2.4.1. Qifi Kit Quantification

To determine quantitative EGFR expression on tumor cells, the Qifi kit (Dako, Heverlee, Belgium) was used according to the manufacturer's protocol. Briefly, tumor cells were harvested, counted, and stained with mouse anti-human EGFR antibody (clone AY13, BioLegend). Afterwards, cells were washed and incubated with FITC-labelled goat anti-mouse F(ab)_2_ fragments (provided with the kit). Cells were analyzed with flow cytometry. Calibration beads were used to make a titration curve to allow the calculation of the absolute number of receptors.

#### 2.4.2. Binding of Anti-EGFR Antibodies

Human carcinoma cells were incubated with anti-human EGFR mAbs or patient plasma at different concentrations for 45′ on ice. After washing, antibodies were detected with PE-labelled polyclonal goat anti-human IgG F(ab)_2_ fragments (AbD Serotec, Kidlington, UK). Cells were analyzed with flow cytometry.

### 2.5. Antibody-Dependent Killing

#### 2.5.1. Antibody-Dependent Phagocytosis

ADCP was performed as described before [38]. Briefly, macrophages were stained with DiO (Molecular Probes Inc, Paisley, UK) in complete RPMI according to the manufacturer's protocol. Tumor cells were stained with eFluor450 (eBioscience) in complete DMEM according to the manufacturer's protocol. Labelled macrophages and tumor cells were cocultured in an effector to target E : T ratio of 5 : 1 in the presence of anti-EGFR mAbs or plasma. After 4 h of coculture, cells were harvested, and percentages of remaining tumor cells were determined by flow cytometry. Percentage killing was calculated by 100—percentage of remaining tumor cells.

#### 2.5.2. Antibody-Dependent Cellular Cytotoxicity

ADCC was performed by incubation of NK cells with tumor cells in an E : T ratio of 10 : 1 in the presence of anti-EGFR mAbs. After 24 h of coculture, plates were carefully washed, and a three-hour cell titer blue (CTB) assay (Promega, Leiden, the Netherlands) was performed according to the manufacturer's protocol. Readout was performed on a Bio-Rad model 680 microplate reader (Bio-Rad, Hercules, CA). Percentage killing was calculated by 100—percentage of remaining tumor cells.

### 2.6. Cell Proliferation and Migration

#### 2.6.1. Cell Proliferation

Tumor cells were seeded in 96 well plates and left to adhere overnight. On day 0, the medium was replaced with complete DMEM containing anti-EGFR mAbs, and cells were cultured for 72 h. Cell viability was analyzed by a CTB assay.

#### 2.6.2. Cell Migration

7 × 10^4^ tumor cells were seeded into culture inserts designed for *in vitro* migration/wound healing experiments (Cat no: 80209, Ibidi, Martinsried, Germany). Culture inserts were placed in 24 well *μ*-plates (Ibidi) and left to adhere overnight. On day 0, inserts were removed, and 1 ml complete DMEM containing anti-EGFR mAbs was added. Gap closure was analyzed over time with an Olympus IX81-ZDC live cell imager.

### 2.7. Statistical Analysis

Graphs were produced, and statistical analysis was performed in GraphPad Prism 8. Bars depicted in the graphs represent the mean ± standard error of the mean (SEM). Differences in data were analyzed with either Student's *t*-tests—in case of two groups—or two-way ANOVA tests followed by Bonferroni's multiple comparison tests—in case of more than two groups. *P* values <0.05 were considered statistically significant.

## 3. Results

### 3.1. Colorectal Cancer Patients Have Increased Numbers of EpCAM + EGFR + Cells in Their Circulation

**Figure 1:**
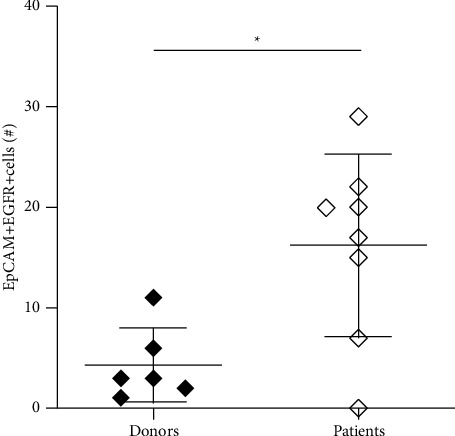
Detection of EpCAM + EGFR + cells in peripheral blood samples. Absolute numbers of EpCAM + EGFR + cells in blood samples of healthy donors (open diamonds) and colorectal cancer patients (closed diamonds). *∗*=*p* < 0.05.

We optimized the protocol based on the detection of CTCs in blood by flow cytometry to be able to detect EGFR + CTCs [39]. Healthy donor blood samples were spiked with no, 50, 100, or 500 HT29 cells. After isolation of the PBMC layer and enrichment by EpCAM beads, samples were stained for the presence of EpCAM + EGFR + cells. Tumor cell recovery was more than 85% in samples spiked with as low as 100 HT29 cells (Supplementary Figure 1(a)). Additionally, we investigated whether cetuximab binding interferes with the detection of CTCs, as patients will be treated with cetuximab prior to surgery in a clinical setting. We confirmed that tumor cells that had been preincubated with cetuximab showed similar binding to the anti-EGFR detection antibody (Supplementary Figure 1(b)), indicating that both anti-EGFR antibodies bind to different epitopes and therefore do not interfere with each other. Next, the number of EpCAM + EGFR + cells in blood samples of patients with metastatic colorectal cancer was determined. Increased numbers of EpCAM + EGFR + cells were detected in the majority of the patients compared to healthy donors (Figure 1).

### 3.2. Similar Opsonization of EGFR-Expressing Tumor Cells by Cetuximab, Panitumumab, and Zalutumumab

**Figure 2:**
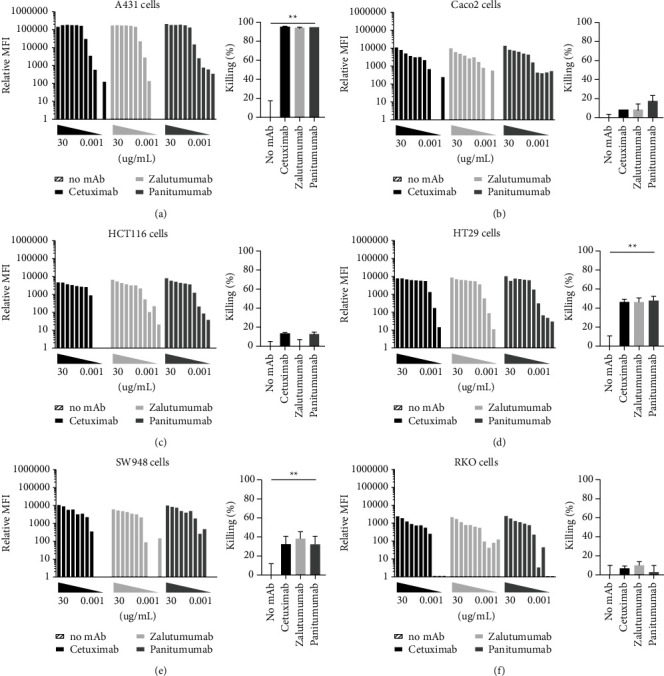
Binding of anti-EGFR mAbs to tumor cell lines and subsequent ADCP by macrophages. The anti-EGFR mAbs cetuximab (black bars), zalutumumab (light grey bars), and panitumumab (dark grey bars) were used for opsonization (a) of (A) A431, (B) Caco2, (C) HCT116, (D) HT29, (E) SW948, and (F) RKO cells. Concentrations were 30–10–5–2.5–1–0.5–0.1–0.05–0.01–0.005–0.001 *μ*g/ml. MFI = mean fluorescence intensity. Phagocytosis (b) of (A) A431, (B) Caco2, (C) HCT116, (D) HT29, (E) SW948, and (F) RKO cells by human monocyte-derived macrophages in the presence of 0.1 *μ*g/ml cetuximab (black bars), zalutumumab (light grey bars), and panitumumab (dark grey bars). Tumor cell killing was analyzed by flow cytometry. The percentage killing shown is relative to the no antibody control. Bars represent mean ± SEM. ^*∗*^=*p* < 0.05, ^*∗∗*^=*p* < 0.01.

Although approximately 80% of colorectal cancer patients have EGFR-expressing tumors, the level of EGFR expression can differ between tumors. Therefore, we determined EGFR expression on different epithelial tumor cell lines used in our assays. The epidermoid carcinoma cell line A431 had high EGFR overexpression (>300,000 molecules per cell) (Table 1). The colorectal carcinoma cell lines Caco2, HCT116, HT29, and SW948 had similar EGFR expression, ranging from 20,000 to 40,000 molecules per cell. The colorectal carcinoma cell line RKO had about 10 times less EGFR expression, whereas the colorectal carcinoma cell line SW620 hardly expressed EGFR. Tumor cells were opsonized by different concentrations of the anti-EGFR mAbs in a dose-dependent manner. In most cases, 0.05 *μ*g/ml anti-EGFR mAbs was sufficient for >90% saturation (Figure 2(a)). Overall, no major differences were observed between the different anti-EGFR mAbs.

### 3.3. Macrophages Efficiently Induced ADCP at Low Anti-EGFR Antibody Concentrations

We previously demonstrated that antibody treatment could prevent surgery-induced metastasis development, which was mediated through ADCP in a rat model. Therefore, we investigated whether the available anti-EGFR mAbs induced ADCP of tumor cells by macrophages. A431 cells, which have very high EGFR expression, were killed extremely efficiently by macrophages in the presence of a low concentration (0.1 *μ*g/ml) of anti-EGFR mAb (Figure 2(b)). When HT29 cells were cocultured with macrophages in the presence of 0.1 *μ*g/ml cetuximab, ∼50% tumor cell killing was induced. Similar results were obtained with SW948 cells as a target, whereas RKO cells, with very low EGFR expression, were not eliminated. Similarly, macrophages were not able to kill Caco2 cells or HCT116 cells, despite intermediate EGFR expression.

No apparent difference in ADCP induction was observed between the different anti-EGFR mAbs, although panitumumab is of the IgG2 isotype, which has a lower affinity for IgG Fc-receptors (Fc*γ*R). When ADCC assays with either HCT116 or HT29 cells and NK cells were performed in the presence of cetuximab or zalutumumab, efficient ADCC was induced (Supplementary Figure 2; data not shown), while this was not the case in the presence of panitumumab. Thus, all three anti-EGFR mAbs efficiently induced ADCP by macrophages, but only antibodies of the IgG1 isotype induced ADCC by NK cells.

### 3.4. Proliferation and Migration Were Not Affected by Low Doses of Anti-EGFR Antibodies

**Figure 3:**
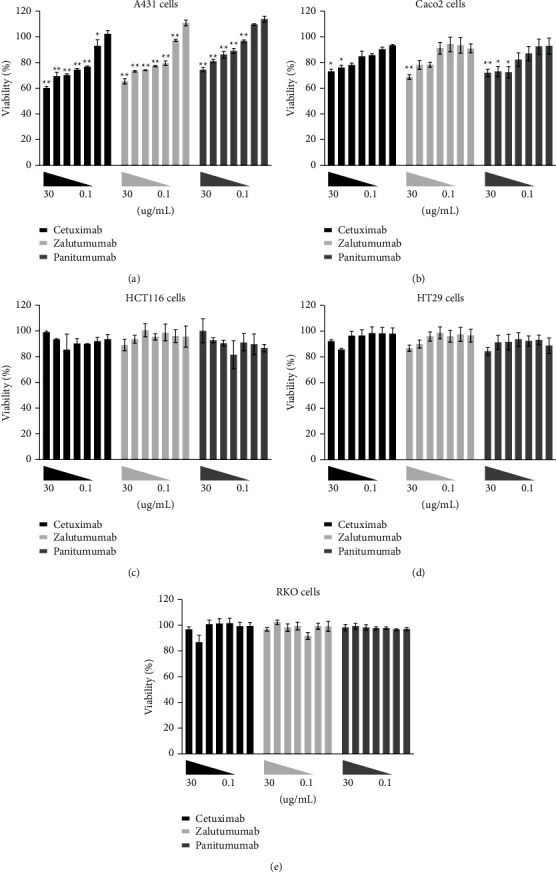
Proliferation of tumor cells in the presence of anti-EGFR mAbs. Proliferation of (a) A431, (b) Caco2, (c) HCT116, (d) HT29, and (e) RKO cells in the presence of different concentrations of the anti-EGFR mAbs cetuximab (black bars), zalutumumab (light grey bars), and panitumumab (dark grey bars). Concentrations were 30–10–5–2.5–1–0.5–0.1 *μ*g/ml. Bars represent mean ± SEM. ^*∗*^=*p* < 0.05, ^*∗∗*^=*p* < 0.01 compared to the lowest concentration.

Healthy colon cells express EGFR as well, albeit at lower levels compared to most colorectal tumors. Nonetheless, treatment with anti-EGFR antibodies could interfere with wound healing through inhibition of EGFR signaling, which would preclude perioperative use. Therefore, the direct effect of anti-EGFR antibodies on cell proliferation and migration was investigated, as both processes are essential for effective wound healing. First, the direct effect of anti-EGFR antibodies was explored on tumor cells without mutations in EGFR signaling pathways (referred to as EGFR wild-type). The proliferation of A431 cells–with high EGFR overexpression–was reduced in the presence of 1 *μ*g/ml or more anti-EGFR mAbs (Figure 3(a)). In contrast, the proliferation of Caco2 cells was somewhat reduced in the presence of more than 5 *μ*g/ml (Figure 3(b)). As expected, the presence of anti-EGFR mAbs did not affect the proliferation capacity of HCT116 cells, HT29 cells, and RKO cells, which harbor mutations in EGFR signaling pathways (Figures 3(c)–3(e)).

**Figure 4:**
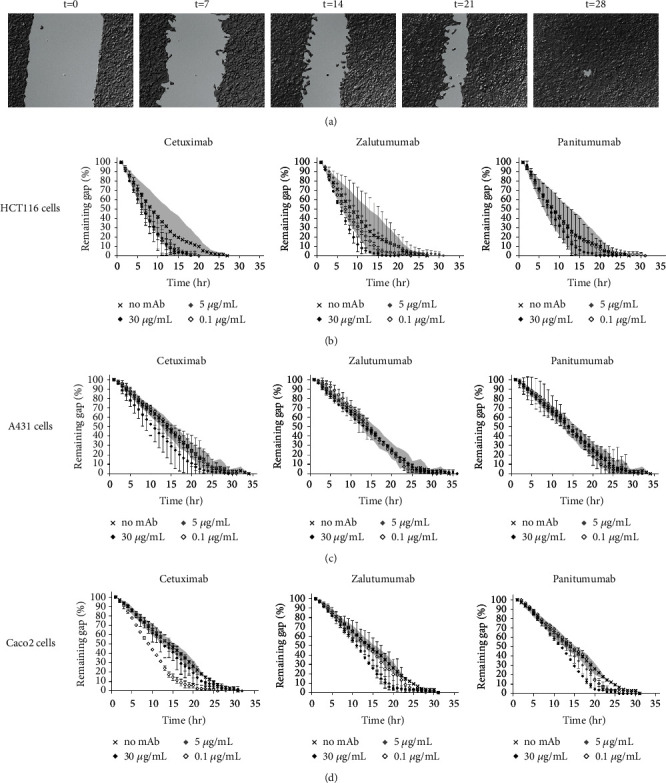
Migration of tumor cells in the presence of anti-EGFR mAbs. (a) Bright-field images of a time lapse video, showing migration of A431 cells over time. Time points are indicated (hours). (b–d) Areas of the gaps were measured at every time point and closure relative to time point *t* = 0 (100%) was calculated. Sizes of the gaps over time, when (b) HCT116, (c) A431, and (d) Caco2 cells were cultured in the presence of different concentrations of the anti-EGFR mAbs cetuximab (left columns), zalutumumab (middle columns), and panitumumab (right columns), were determined. Grey areas represent SEM of the no antibody control (×). Concentrations were 30 *μ*g/ml (black squares), 5 *μ*g/ml (grey squares), and 0.1 *μ*g/ml (white squares).

Next, culture inserts were used to mimic wound healing of a gap (Figure 4(a)). Untreated HCT116 cells were able to completely close the gap in approximately 27 h (Figure 4(b)). No significant differences were observed in the presence of the different anti-EGFR mAbs at concentrations up to 30 *μ*g/ml. Interestingly, neither A431 cells nor Caco2 cells, which were affected in their proliferation capacity in the presence of high concentrations of anti-EGFR mAbs, were affected in their ability to close the gap (Figures 4(c)–4(d)).

### 3.5. Macrophages Efficiently Induced ADCP in the Presence of 0.1% Plasma of Cetuximab-Treated Patients

**Figure 5:**
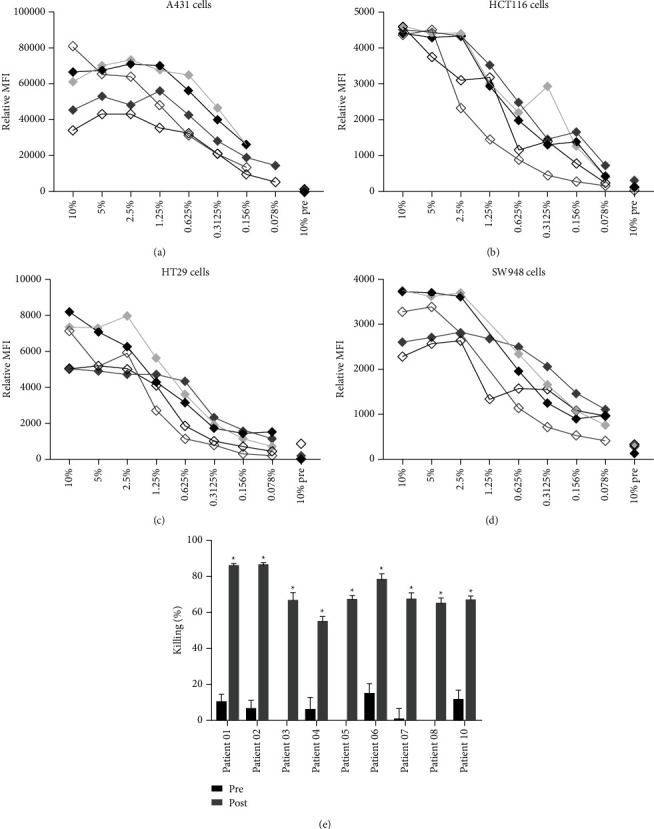
Incubation of tumor cells with plasma from cetuximab-treated patients and subsequent ADCP by macrophages. (a) A431, (b) HCT116, (c) HT29, and (d) SW948 cells were incubated with diluted plasma from cetuximab-treated patients. Dilutions ranged from 10% to 0.078%. 10% plasma that had been obtained prior to treatment was used as the negative control. Binding of cetuximab was determined with flow cytometry. Titration curves of plasma from five different patients are shown. MFI = mean fluorescence intensity. (e) Human monocyte-derived macrophages were incubated with A431 cells in the presence of 0.1% plasma from patients pre- and postcetuximab infusion. Tumor cell killing was analyzed by flow cytometry. The percentage killing shown is relative to the no plasma control. Bars represent mean ± SEM. ^*∗*^=*p* < 0.01.

Patients with metastatic KRAS wild-type colorectal carcinoma with primary tumors originating in the left-sided colon can be treated with cetuximab or panitumumab monotherapy. When plasma from patients treated with cetuximab (obtained four hours after infusion) was used to opsonize tumor cell lines, 2.5% plasma was sufficient for maximal saturation of EGFR on different cell lines (Figures 5(a)–5(d)). Subsequently, we investigated whether the cetuximab concentration in plasma from cetuximab-treated patients was sufficient to induce tumor cell killing via ADCP. The cetuximab concentration in plasma ranged from 100 to 250 *μ*g/ml as determined by ELISA (data not shown). No titer was detected in plasma samples obtained before cetuximab treatment. Incubation of A431 cells and human monocyte-derived macrophages in the presence of 0.1% postcetuximab plasma (but not precetuximab plasma), resulted in efficient ADCP (Figure 5(e)). Some interpatient heterogeneity in the efficiency of tumor cell killing, ranging from 55–85%, was observed, which did not correlate with the cetuximab concentration in plasma.

## 4. Discussion

The presence of CTCs correlates with poor survival in patients with colorectal cancer, even after resection of the primary tumor/liver metastases with curative intent [11, 12]. Perioperative treatment that eliminates CTCs may significantly improve patient outcomes, as perioperative chemotherapy has shown limited clinical efficacy [23]. In this study, we demonstrated that EGFR represents a suitable target for preoperative antibody treatment. The presence of EGFR-expressing CTCs was shown in the majority of colorectal cancer patients. Treatment with anti-EGFR mAbs induced potent ADCP at a concentration at which no direct effects on proliferation and migration were observed, which could hamper wound healing.

Despite multiple studies on (combinations of) treatment options [40], clinical guidelines for the treatment of metastatic colorectal cancer have not changed significantly over the past years [7, 41–43]. Currently, the anti-EGFR mAbs cetuximab and panitumumab are used in the treatment of metastatic KRAS wild-type colorectal cancer patients [3, 4, 7]. These mAbs function as antagonists for EGFR, resulting in inhibition of proliferation [24, 25]. Additionally, these mAbs recruit immune cells, which can then induce ADCP or, in the case of cetuximab, ADCC, resulting in tumor cell killing [29, 44, 45]. However, only 10% of patients with metastatic colorectal cancer showed clinical responses after monotherapy with anti-EGFR mAbs [46]. Response rates could be enhanced to 20–30% by combining anti-EGFR antibody treatment with chemotherapy [46]. These poor response rates may be explained by mutations in components of EGFR signaling pathways, such as KRAS and BRAF. Consequently, these mutations result in continuous activation of the EGFR signaling pathway, irrespective of EGF binding [47, 48]. When the effect of anti-EGFR mAbs was analyzed in patients with metastatic colorectal cancer with wild-type KRAS only, clinically relevant response rates increased to approximately 60% [49, 50]. Therefore, anti-EGFR antibody treatment was only indicated when patients had no mutations in KRAS or BRAF until recently, combination treatment of cetuximab with encorafenib and binimetinib was approved for the treatment of patients with metastatic colorectal cancer harboring a BRAF V600 E mutation [42]. However, as anti-EGFR antibody treatment induces ADCP of human colorectal cancer cells by macrophages, independent of mutations in EGFR signaling pathways, mutations in KRAS or BRAF are not expected to negatively impact the efficacy of preoperative antibody treatment to eliminate CTCs. In contrast, EGFR expression on CTCs will likely be a limiting factor. Tumor cells with high EGFR expression were efficiently phagocytosed by macrophages, even in the presence of low anti-EGFR mAb concentrations. Tumor cells with low EGFR expression were not phagocytosed, even when high anti-EGFR mAb concentrations were added.

In addition to inhibition of tumor cell proliferation and induction of tumor cell killing via activation of immune cells, mAbs can induce complement-dependent cytotoxicity (CDC) [29, 44, 45]. The role of CDC in patients has not yet been completely elucidated. A correlation between polymorphisms in the C1QA gene and clinical outcome in patients with follicular lymphoma treated with the anti-CD20 mAb rituximab suggests a potential role for antibody-induced CDC [51]. Patients with chronic lymphocytic leukemia treated with the anti-CD20 mAb ofatumumab showed a higher response rate compared to rituximab, possibly due to enhanced activation of the complement pathway [52]. It has been demonstrated that anti-EGFR mAbs had poor capacity to induce CDC of EGFR-expressing tumor cell lines *in vitro*, rendering a role for cetuximab-induced CDC in patients less likely [53]. Modulation of antibody binding and valency was shown to enhance anti-EGFR mAb-induced CDC. Therefore, antibody engineering may provide interesting options to further enhance antitumor immune responses.

No major differences were observed in phagocytosis of tumor cells by macrophages in the presence of cetuximab, panitumumab, or zalutumumab. However, while both cetuximab and zalutumumab-induced ADCC of tumor cells by NK cells, panitumumab did not. This is likely due to differences in Fc*γ*R expression on macrophages and NK cells. NK cells only express Fc*γ*RIIIa, which binds with high affinity to IgG1 antibodies, such as cetuximab and zalutumumab. However, IgG2 antibodies, such as panitumumab, bind only with low affinity to Fc*γ*RIIIa [54, 55]. Macrophages express Fc*γ*RI and Fc*γ*RIIa, in addition to Fc*γ*RIIIa. Therefore, it is likely that phagocytosis of tumor cells induced by IgG2 mAbs is mediated via either Fc*γ*RI or Fc*γ*RIIa. Consequently, it is beneficial to use antibodies of the IgG1 isotype in anticancer treatment. In this way, both macrophages and NK cells can be recruited as effector cells for the elimination of tumor cells.

ADCP of Caco2 cells and HCT116 cells were ineffective in spite of EGFR expression levels similar to those of other cell lines that were phagocytosed. Several studies demonstrated immune evasion by tumor cells through the expression of a multitude of receptors, such as CD47 and programmed cell death ligand 1 (PD-L1). High CD47 expression on tumor cells acts as a “do not eat me” signal through binding to the inhibitory receptor SIRP-*α* on macrophages [56]. PD-L1 was originally described as a signaling molecule on tumor cells to prevent T cell-mediated tumor cell killing through interaction with PD-1 [57]. More recently, the interaction between PD and L2 on tumor cells and repulsive guidance molecule B (RGMb) on macrophages was observed that prevented macrophage-mediated tumor cell killing [58]. Thus, tumor cells can evade both adaptive and innate immune responses by inhibiting the effector functions of T cells and macrophages, respectively. Therefore, combining tumor-targeting mAbs with checkpoint inhibitors blocking these and other inhibitory receptors may improve antibody treatment efficacy.

We have previously shown that Kupffer cells and, to a lesser extent, monocytes are potent inducers of phagocytosis of tumor cells present in the bloodstream [32, 59, 60]. Therefore, we propose a novel antibody-based treatment strategy in which anti-EGFR mAbs are administered shortly before surgery to eliminate CTCs. However, it is essential that wound healing is not affected by this treatment strategy since leakage of the anastomosis may result in major infectious complications due to colonization of the colon by abundant microflora. Metastatic colorectal cancer patients amenable for anti-EGFR mAb treatment currently receive relatively high doses (250–500 mg/m^2^ of cetuximab or 6 mg/kg of panitumumab), which is aimed to block EGF binding to EGFR. These high antibody concentrations are presumably not needed to eliminate CTCs by the immune system, as efficient ADCP was observed with 0.1% plasma of cetuximab-treated patients. Additionally, it was demonstrated in an *in vivo* xenograft model that low antibody concentrations were sufficient to eliminate CTCs, independent of KRAS mutations [61]. Only minor inhibition in the proliferation capacity of cell lines with a wild-type EGFR signaling pathway was shown in the presence of low anti-EGFR mAb concentrations. In contrast, there was no effect on migratory capacity. As these two essential processes in wound healing were only marginally affected, we anticipate that healing of the anastomosis will be minimally influenced by preoperative infusion of a low dose of cetuximab.

In conclusion, colorectal cancer patients may benefit substantially from preoperative treatment with tumor-targeting mAbs, as this may lead to the elimination of tumor cells in the circulation by effector cells, such as macrophages in the liver and NK cells. We demonstrated that the current anti-EGFR antibody treatment dose can probably be significantly lowered for this purpose since diluted patient plasma was sufficient to completely opsonize tumor cells and induce efficient ADCP. Lower anti-EGFR mAb concentrations did not influence processes involved in wound healing. Therefore, we propose that colorectal cancer patients can be safely treated preoperatively with a low dose of anti-EGFR antibodies to eliminate CTCs, which may ultimately prevent metastasis development.

## Figures and Tables

**Table 1:** EGFR expression.

Cell line	EGFR molecules (#)
A431	>300,000
Caco2	∼20,000
HCT116	∼30,000
HT29	∼40,000
SW948	∼30,000
RKO	∼4,000
SW620	∼200

## Data Availability

All data relevant to this study are included within the article and supplementary files and are available from the corresponding author upon reasonable request.

## Abbreviations

ADCC: Antibody-dependent cellular cytotoxicity
ADCP: Antibody-dependent cellular phagocytosis
CDC: Complement-dependent cytotoxicity
CTB: Cell titer blue
CTC: Circulating tumor cell
EGF: Epidermal growth factor
EGFR: Epidermal growth factor receptor
Fc*γ*R: IgG Fc-receptor
FCS: Fetal calf serum
mAb: Monoclonal antibody
M-CSF: Macrophage colony-stimulating factor
NK: Natural killer
PBMC: Peripheral blood mononuclear cell
PD-L1: Programmed cell death ligand 1
RGMb: Repulsive guidance molecule B
VEGF: Vascular endothelial growth factor.

## Additional Points

This study provides robust data for the use of clinically available antibodies in a new context to improve the survival of colorectal cancer patients. Moreover, the authors anticipate that this novel therapeutic strategy may be expanded to other types of cancers.

## Supplementary Materials

Supplementary Materials
*Supplementary figure 1.* Validation of CTC detection protocol. (A) Healthy donor blood samples were spiked with 0, 50, 100 or 500 HT29 cells (left to right) and stained for the presence of EpCAM + EGFR + cells. (B) A431 cells were stained with PE-labeled anti-EGFR antibody without (left) or with (middle) pre-incubation with Alexa488-labeled cetuximab. Overlay of anti-EGFR-PE staining (right) (blue = unstained, red = anti-EGFR-PE, orange = anti-EGFR-PE with cetuximab pre-incubation). *Supplementary figure *2. IgG1 antibodies efficiently induce ADCC by NK cells. Human NK cells were incubated with HT29 cells in the presence of various concentrations of cetuximab (black bars), zalutumumab (light grey bars) and panitumumab (dark grey bars). Tumor cell killing was analyzed with a CTB assay. Concentrations were 30 – 10 – 5 – 1 – 0.1 µg/ml. The percentage killing shown is relative to the no antibody control. Bars represent mean ± SEM. ^*∗*^=*p* < 0.01.
