# Supplementary material for: Epidermal Growth Factor Receptor as Target for Perioperative Elimination of Circulating Colorectal Cancer Cells
Source: J Oncol. 2022 Jan 7;2022:3577928. doi: 10.1155/2022/3577928 (PMC8759909; doi:10.1155/2022/3577928)
Supplement: Supplementary Materials — Supplementary figure 1. Validation of CTC detection protocol. (A) Healthy donor blood samples were spiked with 0, 50, 100 or 500 HT29 cells (left to right) and stained for the presence of EpCAM + EGFR + cells. (B) A431 cells were stained with PE-labeled anti-EGFR antibody without (left) or with (middle) pre-incubation with Alexa488-labeled cetuximab. Overlay of anti-EGFR-PE staining (right) (blue = unstained, red = anti-EGFR-PE, orange = anti-EGFR-PE with cetuximab pre-incubation). Supplementary figure 2. IgG1 antibodies efficiently induce ADCC by NK cells. Human NK cells were incubated with HT29 cells in the presence of various concentrations of cetuximab (black bars), zalutumumab (light grey bars) and panitumumab (dark grey bars). Tumor cell killing was analyzed with a CTB assay. Concentrations were 30 – 10 – 5 – 1 – 0.1 µg/ml. The percentage killing shown is relative to the no antibody control. Bars represent mean ± SEM. ∗=p < 0.01. [file 3577928.f1.docx]

# **Supplementary figures**

## **Supplementary figure 1**


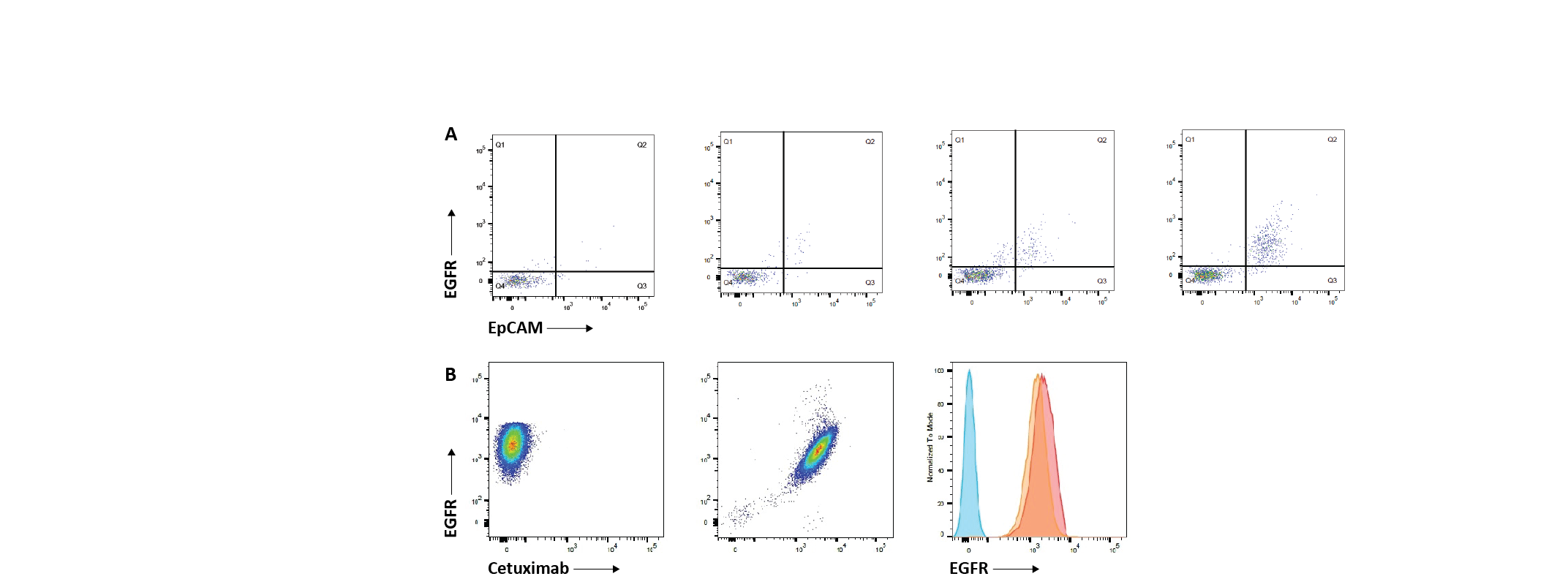


**Validation of CTC detection protocol.** (**A**) Healthy donor blood samples were spiked with 0, 50, 100 or 500 HT29 cells (left to right) and stained for the presence of EpCAM+EGFR+ cells. (**B**) A431 cells were stained with PE-labeled anti-EGFR antibody without (left) or with (middle) pre-incubation with Alexa488-labeled cetuximab. Overlay of anti-EGFR-PE staining (right) (blue = unstained, red = anti-EGFR-PE, orange = anti-EGFR-PE with cetuximab pre-incubation).

## **Supplementary figure 2**


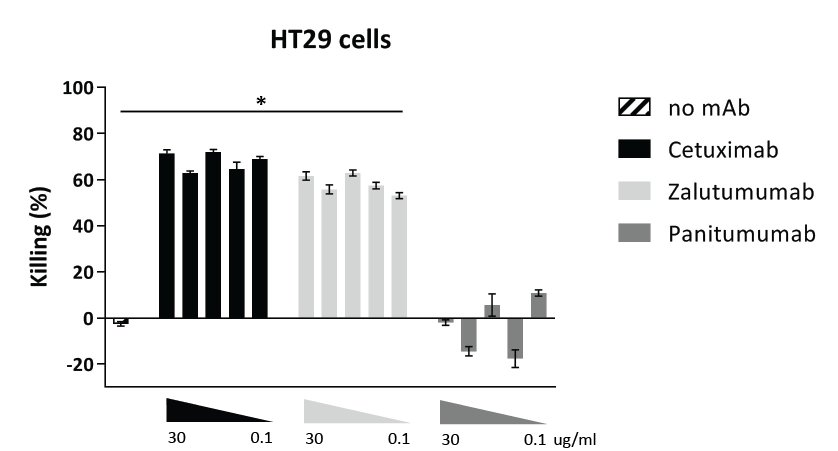


**IgG1 antibodies efficiently induce ADCC by NK cells.** Human NK cells were incubated with HT29 cells in the presence of various concentrations of cetuximab (black bars), zalutumumab (light grey bars) and panitumumab (dark grey bars). Tumor cell killing was analyzed with a CTB assay. Concentrations were 30 – 10 – 5 – 1 – 0.1 µg/ml. The percentage killing shown is relative to the no antibody control. Bars represent mean ± SEM. * = p < 0.01.
